# Application of Genomics to Understand Salt Tolerance in Lentil

**DOI:** 10.3390/genes12030332

**Published:** 2021-02-25

**Authors:** Ruwani Dissanayake, Noel O.I. Cogan, Kevin F. Smith, Sukhjiwan Kaur

**Affiliations:** 1 Agriculture Victoria, AgriBio, Centre for AgriBioscience, Bundoora, VIC 3083, Australia; ruwani.dissanayake@agriculture.vic.gov.au (R.D.); noel.cogan@agriculture.vic.gov.au (N.O.I.C.); 2 Faculty of Veterinary and Agricultural Sciences, The University of Melbourne, Parkville, VIC 3010, Australia; kfsmith@unimelb.edu.au; 3 School of Applied Systems Biology, La Trobe University, Bundoora, VIC 3086, Australia; 4 Agriculture Victoria, Hamilton Centre, Hamilton, VIC 3300, Australia

**Keywords:** lentil, *Lens culinaris*, salt tolerance, genotyping-by-sequencing, genome-wide association study, linkage disequilibrium, haplotypes, pedigree, salt tolerance mechanisms

## Abstract

Soil salinity is a major abiotic stress, limiting lentil productivity worldwide. Understanding the genetic basis of salt tolerance is vital to develop tolerant varieties. A diversity panel consisting of 276 lentil accessions was screened in a previous study through traditional and image-based approaches to quantify growth under salt stress. Genotyping was performed using two contrasting methods, targeted (tGBS) and transcriptome (GBS-t) genotyping-by-sequencing, to evaluate the most appropriate methodology. tGBS revealed the highest number of single-base variants (SNPs) (c. 56,349), and markers were more evenly distributed across the genome compared to GBS-t. A genome-wide association study (GWAS) was conducted using a mixed linear model. Significant marker-trait associations were observed on Chromosome 2 as well as Chromosome 4, and a range of candidate genes was identified from the reference genome, the most plausible being potassium transporters, which are known to be involved in salt tolerance in related species. Detailed mineral composition performed on salt-treated and control plant tissues revealed the salt tolerance mechanism in lentil, in which tolerant accessions do not transport Na^+^ ions around the plant instead localize within the root tissues. The pedigree analysis identified two parental accessions that could have been the key sources of tolerance in this dataset.

## 1. Introduction

Soil salinity is identified as the second most significant abiotic stress after drought and in lentil (*Lens culinaris* Medik.), salt toxicity typically causes between 20% and 100% reduction in plant growth and seed yield [1,2]. In Australia, c. 32 million ha of agricultural land is recognized as highly saline [3,4], therefore, lentil cultivation is limited to an area of c. 360,120 ha with an annual production of 533,755 tonnes, thus making Australia the third largest producer of lentil in the world [5]. Salt stress inhibits plant growth via imposing Cl^-^ ion-specific toxicity, creating an imbalance of ions (Ca^2+^, K^+^, Na^+^), thereby enforcing osmotic pressure, which interferes with soil water extraction. This results in severe distress to the plant’s morpho-physiological and biochemical features and significantly reduces crop yield [6]. Lentil is considerably more sensitive to salt than other grain crops, with more than 90% of yield loss recorded at electrical conductivity (EC) of 3 dS/m compared to barley (*Hordeum vulgare* L.; 10% yield loss at EC = 6.6 dS/m), wheat (*Triticum aestivum* L.; 10% yield loss at EC = 4.9 dS/m), and canola (*Brassica napus* L.; 10% yield loss at EC = 7.3 dS/m) [7,8].

Soil reclamation is one of the strategies used to improve the properties of sodic soils. However, such methods may not be cost-effective for growers in these areas [2,8]. Plants also have defence mechanisms to avoid salt toxicity [9,10]. Ion-homeostasis is one of the well-studied salt-tolerant mechanisms, which regulates the entry of Na^+^ and K^+^ ions into the cytosol of cells through ion-channels and transporters [10,11,12]. The opening of these porters is regulated by multiple proteins/genes, such as translocating enzymes, Salt Overly Sensitive (*SOS*) proteins and high-affinity potassium transporter (*HKT*) genes [9,13]. Many salt-tolerant grain legumes also produce osmolytes/compatible solutes such as organic acids, sugars, and nitrogen-containing compounds, to maintain the hydrophilic nature of the cell membranes and cell structures [14,15]. Soil salinity also causes variation in the production of antioxidants and hormones (e.g., Abscisic acid (ABA) and ethylene (ET)), enabling the exclusion of unwanted chemicals, regulation of other metabolic changes, and stress-responsive genes under salt stress [12,16]. However, little is known about the salt tolerance mechanism in lentil except for a few studies on understanding the variation in Na^+^ and K^+^ ion concentrations [17,18,19].

Therefore, an alternative approach is to understand the genetic basis of salt responsive traits and develop salt-tolerant cultivars. Many other grain legumes and higher plants have attempted this approach for abiotic and biotic characteristics, including soil salinity [1,20,21]. Recently, GWAS has been used as a powerful tool to dissect the genetic basis of many phenotypic traits using genetically diverse populations [22,23]. In general, this approach statistically confirms the association strength between a genotype and phenotype and provides information on molecular markers, alleles and candidate genes that contribute to specific traits [21,23]. Currently, it has widely been applied in many plants, including rice (*Oryza sativa* L.) [22,24,25], cotton (*Gossypium hirsutum* L.) [26,27], soybean (*Glycine max* L.) [28] and maize (*Zea mays* L.) [29], for identifying precise chromosomal locations to define many potential candidate genes for salt stress. The application of GWAS in lentil and many other grain species has required attention due to the challenges posed by the complexity of their genomes and the lack of detailed genomic resources.

However, rapid improvements in low-cost next-generation sequencing (NGS) and advances in accurate resequencing based on novel genotyping-by-sequencing (GBS) have enabled the GWAS in many grain crops [1,21,28]. Currently, numerous GBS methods are available, broadly classified into genome complexity reduction-based methods (e.g., restriction enzyme-based, transcriptome-based) and target enrichment/target capture (e.g., PCR amplification, molecular inversion probes (MIPS) and hybrid capture) [30]. However, once genomic resources are available, target capture methods are often more popular due to only selectively sequencing genomic regions of interest, typically with increased reliability and robustness [31,32].

Although whole-genome and exome sequencing facilitate the identification of novel genomic variants, the cost per sample and the overall depth of coverage required to influence the genotyping process in plant species with large and complex genomes, making them for some species still cost-prohibitive [33]. The overall scale of data generated can also increasingly pose issues of computational processing and management [34]. Therefore, target-capture-based sequencing has been identified as a valuable approach by many researchers working on animals, human and plant species, focusing on sequencing exons, specific variant regions or functional genes of interest selectively [32]. The assay is comparatively robust and cost-effective and allows for more in-depth sequencing coverage if needed [31,32,35]. Therefore, tGBS is currently widely applied in GWAS, genomic selection (GS) and other functional genomic experiments.

For lentil, many complexity reduction-based GBS approaches (e.g., restriction enzyme-based, transcriptome-based) have been developed and applied [30,36,37]. However, the narrow genetic diversity present within the lentil limits the number of SNPs detected and the efficiency of the approach [36,38]. Therefore, to overcome this constraint, the development of additional GBS methods is required. The generation of the lentil genome sequence [39] has now facilitated pathways to develop novel target-capture-based assays (tGBS) and enable association genetics to improve lentil varieties tolerant/resistant to abiotic and biotic stresses.

In the current study, a collection of Australian lentil accessions was evaluated in an association genetics approach to identify genomic regions relating to salt tolerance. The phenotypic data was attained through conventional and high-throughput phenotyping screens [40]. Two GBS methods, tGBS [41] and GBS-t [42], were used to obtain the genome-wide SNP markers for GWAS analysis. The integration of the phenotypic and genotypic data enabled the GWAS study, allowing the identification of genomic regions/haplotypes and candidate genes responsible for salt tolerance. The salt tolerance mechanism behind lentil was assessed through elemental analysis. Pedigree analysis was performed only on tolerant accessions to understand the main parental accessions contributing to the generation of salt-tolerant accessions and thereby to use in future Australian lentil breeding programs.

## 2. Materials and Methods

### 2.1. Plant Materials and Phenotyping

Seeds for all lentil accessions were attained from the Australian Grains Genebank (AGG), Horsham, VIC, Australia. To obtain leaf material for genotyping, each lentil accession was sown for 2–3 weeks in a climate-controlled glasshouse at 22  ±  2 °C under a 16/8 h (light/dark) photoperiod, at the premises of Agriculture Victoria, Bundoora, VIC, Australia.

The phenotypic data used has previously been described in Dissanayake et al. [40] and also provided in Table S1. To summarize, the study included salt stress screening data from 276 lentil accessions selected from the Pulse Breeding Australia (PBA) lentil breeding program. The phenotypic screening was initially carried-out as a traditional glasshouse-based study (Grains Innovation Park, Horsham, VIC, Australia) with four replicates (Experiment 1). The plant responses to salt toxicity were assessed through salt tolerance scores and shoot dry mass (measured in grams) collected at 10 weeks of post-sowing (Table S1). A wider distribution was observed for salt tolerance score (2.87–7.53), with a mean value of 5.29. For shoot dry mass, a narrow (4.56–5.76) level of distribution was observed with a mean value of 4.93 [40]. To establish the image-based phenotypic assay, a pilot study comprising six lentil genotypes (ILL2024, CIPAL1522 (tolerant); PBA Bolt, PBA Hurricane (moderately tolerant); PBA Ace, and PBA Jumbo2 (intolerant)) with known variation to salt stress was performed using a LemnaTec Scanalyzer 3D (LemnaTec GmbH, Aachen, Germany) plant phenomics platform at Plant Phenomics Victoria (PPV), Bundoora, VIC, Australia (Experiment 2). The optimal salt concentration and growth stage that could distinguish the response of lentil genotypes under salt treatment were determined. A further trial was conducted, with all of the 276 accessions that were screened in Experiment 1 using the image-based phenotyping approach developed in Experiment 2, using a partial replicate design (Experiment 3). A range of phenotypic traits was measured to define the most suitable set of non-destructive traits to study salt stress in lentil (projected shoot area, height, convex hull area, compactness green, and non-green color) (Table S1). All of the traits collected from the traditional phenotypic screen and image-based phenomics screen were normally distributed. Among these traits, traditional phenotypic scores and phenomics color pixels (from the image-based screen) were moderately correlated (*r* = 0.55; *p* < 0.0001) [40].

### 2.2. Probe Designing for Lentil Targeted-GBS (tGBS) Method

Probes for tGBS protocol were designed from the data generated by Malmberg et al. [30]. A total of 182 lentil samples, representing 38 ancestral genotypes (which included salt-tolerant and intolerant sources), were aligned to the lentil reference transcriptome [42]. A total of 231,977 SNP markers were identified from the transcriptome-based GBS method and were used to design probes for the tGBS protocol. These SNPs were compared to the reference genome of lentil CDC Redberry (v1.2) [43] and a set of sites that were uniformly distributed across the genome were selected (Table S2). The flanking sequences of these SNP loci were analyzed against the reference lentil genome to remove any sites with non-specific binding, due to sequence duplication. The resulting filtered flanking sequences were sent to NuGEN^®^ (NuGEN Technologies, Inc., San Carlos, CA, USA) [44] for probe design and synthesis. During this final probe design process, a further set of loci were excluded, ultimately resulting in 65,623 probes that relate to 46,520 targets.

### 2.3. tGBS Library Preparation, Sequencing and Variant Calling

DNA extraction for all lentil accessions was performed using DNeasy^®^ 96 Plant Kit (QIAGEN, Hilden, Germany), following the manufacturer’s instructions. The concentration and quality of DNA were confirmed using a NanoDrop^TM^ UV-Visible spectrophotometer (Thermo-Scientific, Wilmington, DE, USA) at the wavelength ratios of A260/230 and A260/280 nm. The integrity of extracted DNA samples was evaluated using TapeStation 2200 platform with DNA ScreenTape System (Agilent Technologies, Santa Clara, CA, USA), following the manufacturer’s guidelines.

tGBS libraries were constructed using the NuGEN^®^ Allegro TGBS protocol (version 2.0) at quarter reaction volume, starting with 37.5 ng of genomic DNA in 2.5 µL volume (15.0 ng/µL). The reaction volumes of the library preparation were also adjusted accordingly. The concentration, size distribution, and quality of the final library were determined on a TapeStation 2200 platform with HD5000 ScreenTape System (Agilent Technologies, Santa Clara, CA, USA), and the library was paired-end (2 × 150 bp) sequenced using Illumina HiSeq 3000 Sequencing platform (Illumina Inc., San Diego, CA, USA).

Following fastq data generation, quality trimming was done using TrimGalore (version 0.4.4) [45]. The trimmed sequence reads were then aligned to the reference genome sequence of cultivar CDC Redberry (v2.0) [39] using Bowtie2 (version 2.1.9) [46]. Variant calling was performed using SAMtools (version 1.5) [47] that generated the variant call file (VCF). The VCF file was then filtered for a minimum read depth (DP) of 5, maximum missing data of 80%, base quality of 30 (Q30), minimum minor allelic frequency (MAF) of 5%, and heterozygosity of 20%, using VCFtools (version 0.1.16) [48] and the TASSEL software package (Trait Analysis by aSSociation, Evolution, and Linkage; version 5.2.48) [49]. Genetic diversity of the studied lentil population was also analyzed based on the method described in Dissanayake et al. [38] using the StAMPP package [50] and DARwin-6.0.17 software [51] (Figure S1).

### 2.4. Transcriptome-based GBS Library Preparation, Sequencing and Variant Calling

Total RNA extraction, library preparation and quality trimming for GBS-t were performed using the method described in Dissanayake et al. [38]. The remaining high-quality trimmed sequence reads were aligned to the lentil reference genome sequence of cultivar CDC Redberry (v2.0) [39] using Spliced Transcripts Alignment to a Reference (STAR) aligner (version 2.5.4a) [52]. The variant calling and filtering were performed as the pipeline described in Section 2.3.

### 2.5. GWAS, Candidate Genes Identification and Pedigree Haplotype Analysis

Best linear unbiased estimates (BLUE) generated for phenotypic traits were used in the GWAS analysis. The general linear model (GLM) and mixed linear model (MLM) embedded in TASSEL (version 5.2.48) were used to identify the best-fitted model for the lentil population [49]. Bayesian Information Criterion (BIC) in the Genomic Association and Prediction Integrated Tool (GAPIT; version 3.0) was used to identify the optimal number of principal components (PCs) for the GWAS study [53]. Bonferroni-corrected *p*-value of ≤ 0.05 (*p* = 0.05/n; *n* = total SNP markers used, −log_10_ (*p*)) was used as a threshold of significance for the GWAS analysis. Manhattan plots and Quantile-Quantile (QQ) plots were regenerated using the software package RStudio with the CMplot and qqman functions from the CRAN library [54,55,56].

Haplotype blocks were constructed using Haploview (version 4.2) [57]. The formation of the blocks was performed using the confidence intervals method [58], which defined the blocks based on 95% confidence intervals of the D’ values, classifying as strong linkage disequilibrium (LD). The haplotypic variations in tolerant and intolerant accessions (salt tolerance classes identified from the previous study [40], Table S1) were further evaluated based on significant SNPs using TASSEL (version 5.2.48) [49]. Regions identified from marker-trait associations were also assessed using the gene-finding format (GFF) file of CDC Redberry (v2.0) [39] to examine any potential causative genes for salt tolerance in lentil.

Pedigree information for salt-tolerant accessions was attained from the available breeders’ records, Australian genebank passport information, and the Australian pulse breeding information [59] and was visualized using the Helium software package (version 1.19.09.03) [60]. The accessions were color-coded according to the haplotypes identified from the current study.

### 2.6. Understanding Salt Tolerance Mechanism in Lentil Using Elemental Analysis

Lentil accessions for elemental analysis were chosen from the previous image-based phenotyping assay [40]. Briefly, the experiment had both control (0 mmol sodium chloride (NaCl)) and salt-treated (100 mmol) lentil accessions located in adjacent rows within a paired plot design. As described in Section 2.1, plants for salt tolerance were evaluated using digital traits. At the final harvesting stage, manual traits (salt tolerance scores and shoot dry mass) were also measured. Based on the scores and rankings, ten contrasting lentil genotypes categorized as tolerant and intolerant were selected to carry-out mineral assay. Multiple tissue types were collected from salt-treated (upper leaves, lower leaves, stems, roots) and control (only upper leaves) accessions. The tissues were oven-dried at 60 °C for 3 days and grounded into a fine powder using a GenoGrinder (SPEX SamplePrep, USA). The pre-processed samples [61] were used in the determination of the standard suits of elements (Na, K, P, S, Ca, Mg, Cu, Zn, Mn, Fe, B, and Al) by using the inductively coupled plasma optical emission spectroscopy (ICP-OES) instrumentation at Agriculture Victoria, Macleod, VIC, Australia. The respective boxplots for ion concentrations were generated using the software package RStudio with the ggplot2 function from the CRAN library [55]. Statistical significance of each element under different tissue types was calculated using the t-test function in ggpubr and rstatix software packages from the CRAN library [55,62].

## 3. Results

### 3.1. Evaluation of SNP Markers Captured in Novel tGBS Method and GBS-t Method

**Figure 1:**
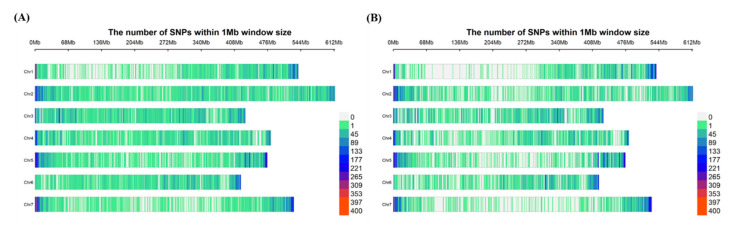
Distribution of SNP markers within 1Mb windows for seven chromosomes (Chr) in lentil (**A**) tGBS method (**B**) GBS-t method.

Attempts were made to identify SNP markers from the tGBS method, using the designed probes and a de novo approach. However, results found that SNP density, and distribution of markers identified from the de novo approach, were much better than the pre-defined design (Table S2). Therefore, the de novo approach was selected as the best pipeline for identifying genome-wide SNP markers for GWAS analysis. VCF files were generated from both Bowtie2-SAMtools and STAR aligner-SAMtools pipelines using a depth of five (DP ≥ 5), and the initial number of SNPs identified was 2,043,680 and 1,614,141 for tGBS and GBS-t methods, respectively. Following the filtration options, a set of 57,344 (tGBS) and 53,186 (GBS-t) high-quality SNP markers remained (Table 1). However, only SNP markers that were located on the seven pseudomolecules were used in the GWAS analysis reducing the numbers further to 56,349 (tGBS) and 52,471 (GBS-t) (Table S3 and S4). The final set of SNP markers were plotted in 1MB (megabase pair) windows across the lentil genome (Figure 1) to compare their distribution of the two GBS methods. The tGBS, marker distribution was more uniform across the chromosomes compared to GBS-t, where lower SNP density was observed on Chr1, Chr5 and Chr7. The biggest interval between SNP markers in tGBS was c. 9.0 Mbp, and for GBS-t, it was c. 22 Mbp. However, in contrast, GBS-t had specific regions where more concentrated marker density could be observed, for example in the telomeric regions of Chr1 and 7. The number of variants detected in each chromosome for each GBS method was summarized in Table S5.

### 3.2. Model Selection for Marker-Trait Association Study

GLM and MLM models were tested for best fit based on the observed and expected *p*-values for the trait. According to the BIC analysis, the optimal number of PCs for each trait was identified as five. Quantile-Quantile plots confirmed the goodness-of-fit and efficiency of the models. The mixed linear model involving PCA and kinship data was identified as the best fit and was therefore used in the association analysis (Figure S2).

### 3.3. Regions Identified for Salt Tolerance Traits Using Different GBS Methods

**Figure 2:**
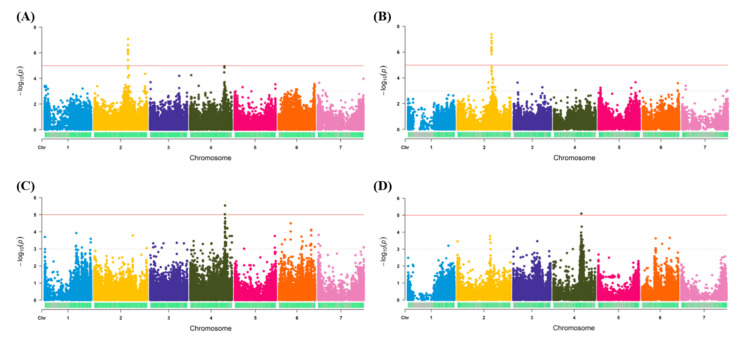
Marker-trait association results for salt tolerance traits. (**A**) and (**B**) represent the plots generated for traditional phenotypic scores. (**C**) and (**D**) represent the plots generated for phenomics screen green color pixels. Plots A and C were generated from the data obtained from tGBS method, while B and D were from GBS-t method. The red line indicated the significant threshold level.

Among the multiple phenotypic traits measured in both traditional and image-based phenomics screens, only traits with strong marker-trait associations were presented in the current study. Therefore, the salt tolerance scores from the traditional phenotypic screen and green color pixels from the image-based approach were used as the phenotypic input data for the GWAS analysis, with both genotyping methods (Table S1; Figure 2). Using the traditional phenotypic scores, significant regions were detected on Chromosome 2 (Chr2) using both GBS methods (Figure 2A,B). For the tGBS method, a total of 12 significant markers (−log_10_ *p*-value ≥ 5) were detected (Figure 2A and Table S6), while for GBS-t, it was 14 significant SNP markers (Figure 2B and Table S6). Upon detailed examination of Chr2, both genotyping methods generated significant associations within the same genomic region of 392–394 Mbp (Figure S3). The tGBS approach also detected significant associations on Chromosome 4 (Chr4), in the region of 400–405 Mbp (Figure 2A and Figure S3).

Using the image-based phenomics data, the region on Chr4 was re-identified using both genotyping methods (Figure 2C,D). For the tGBS method, the marker-trait association was observed in the region of 395–410 Mbp (Chr4), which overlaps with the region identified using the traditional phenotypic scores (Figure 2C and Figure S3). This region was only identified as significant by two markers using this genotyping method (Figure 2C and Table S6). For GBS-t, a small region was detected on Chromosome 4 (358-366 Mbp), however, this was identified by a single significant SNP marker (Figure 2D, Table S6 and Figure S3). Since the genomic regions identified from each GBS method failed to overlap, a broad region of significance was identified using both traditional and phenomics approaches (Chr4_350-410 Mbp) (Figure S3). While not significant, the GBS-t genotyping method did increase in its log scores in the region on Chr2 that overlaps with the traditional phenotypic method (Figure 2D). The association results obtained for other phenotypic traits were summarized in Figure S4.

### 3.4. Haplotype Blocks on Chromosome 2

**Figure 3:**
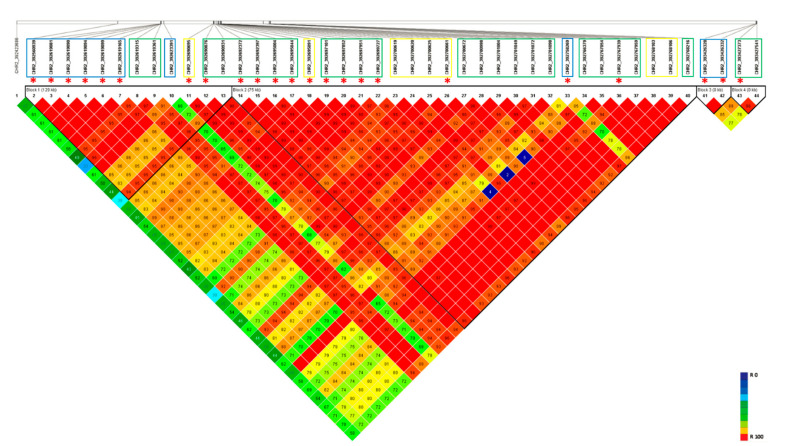
LD heatmap on Chromosome 2 haplotypic region using SNP markers generated from both GBS methods. Markers derived from each GBS method was highlighted using the colored box. Blue: markers from tGBS method; Green: markers from GBS-t method; and Yellow: markers common to both GBS methods. ‘*’ represents the significant marker positions identified in the current study.

The haplotypic region (392–394 Mbp) detected on Chromosome 2 as influencing salt tolerance in lentil was analyzed for LD and haplotypic structure (Figure 3 and Figure S5). The SNP markers generated from both GBS methods were combined to identify haplotype blocks for salt tolerance. Four main haplotypic regions were observed. However, most of the significant marker positions identified on Chr2, using both GBS methods, were clustered into two main blocks, Block 1 (29.0 kb) and Block 2 (75.0 kb).

### 3.5. Candidate Genes Identified for Genomic Regions

The known genes that have been annotated within the associated regions on Chromosome 2 and Chromosome 4 were extracted from the GFF file of the CDC Redberry genome (v2.0). A total of 43 genes were identified on Chromosome 2 haplotypic region (Chr2_392–394 Mbp; Table S7). Among them, a high-affinity potassium transporter gene (Lcu.2RBY.2g061250) was identified as a high priority candidate for further investigation over its association with salt tolerance in lentil. For Chromosome 4, 1195 genes were identified for the broad haplotypic region (Chr4_350-410 Mbp; Table S7). Among them were, potassium transporter, *SOS1*, vacuolar protein sorting-associated protein and several other transporter genes (e.g., calcium transporting ATPase, auxin transporters, phosphorous transporters, iron transporters, and sulphate ion transporters) present that, based on annotation could be potentially associated with salt tolerance in lentil. Table S7 has summarized the genes related to both haplotypic regions.

### 3.6. Haplotype Variation on Chromosome 2

A total of 81 lentil accessions that had been clearly categorized as tolerant or intolerant (Table S1; 42 tolerant lentil accessions, 39 intolerant lentil accessions) were evaluated for their corresponding haplotypes within the significantly detected genomic regions to dissect the inheritance of the favorable alleles for salt tolerance in lentil (Table S8). For the tolerant accessions, three main haplotypes (Hap1, Hap2, and Hap1/2) were observed, and the majority of the accessions were classified under Hap1. For the intolerant genotypes, most of the accessions were identified as Hap2 (Table S8). Table 2 indicated how the favorable alleles presented in tolerant and intolerant accessions for the major genomic region were observed on Chromosome 2. Any genotype with missing data or heterozygous allele calls were excluded from Table 2.

### 3.7. Pedigree Analysis

**Figure 4:**
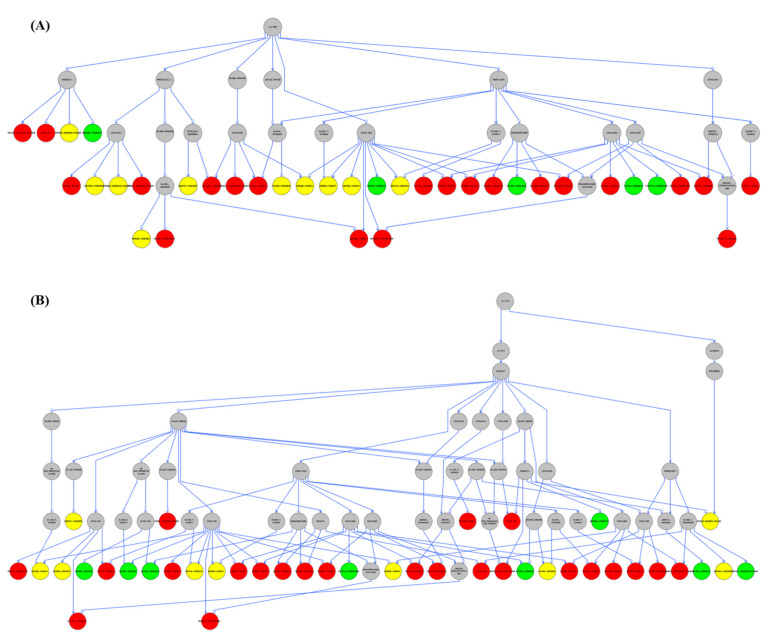
Pedigree analysis on tolerant lentil accessions (**A**) ILL7685 as the parental source (**B**) ILL1719 as the parental source. Salt tolerant accessions were color-coded according to the haplotypes identified in the current study; Red: Hap1, Green: Hap2, and Yellow: Hap1/2.

Pedigree analysis was also performed on the tolerant accessions to identify potential parental accessions contributing to salt tolerance in the lentil population (Table S9). Out of 42 tolerant lentil accessions, 36 were identified as sharing a common ancestor; ILL7685 (Figure 4A and Figure S6). The accessions that did not have ILL7685, were identified as containing the parent; ILL1719, as the potential second source of salt tolerance for five of the tolerant lentil accessions (Figure 4B and Figure S7). There was, however, a single accession that failed to have sufficient pedigree information to identify its potential parents (Figures S6 and S7).

### 3.8. Understanding Salt Tolerance Mechanism in Lentil

**Figure 5:**
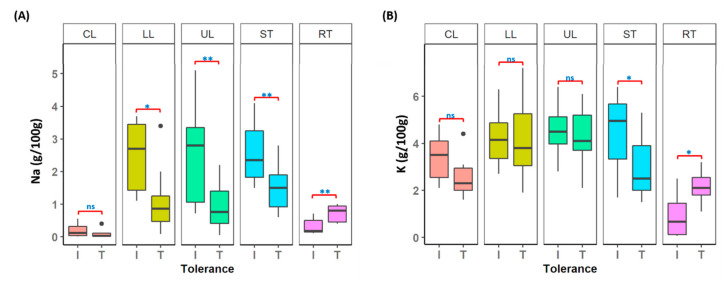
Elemental analysis on different tissue types (CL: leaves from control plants; UL: upper leaves; LL: lower leaves; ST: stems and RT: roots from salt-treated plants) collected from lentil accessions. I: Intolerant accessions and T: Tolerant accessions (A) Na (B) K; ns (not significant): *p* > 0.05, * *p* < 0.05 and ** *p* < 0.001.

Multiple plant tissues harvested from individual plants were used for macro- and micro-nutrient analysis to understand the variation in chemical composition in tolerant and intolerant lentil accessions, under control and salt-treated conditions. Among the 12 elements measured (Figure S8), the two main elements (Na and K) known to be associated with the salt tolerance mechanism were illustrated in Figure 5. In tolerant accessions (T), a lower level of Na concentration was maintained in both leaves (LL and UL) and stems (ST), compared to intolerant (I) accessions. However, the Na concentration in roots (RT) of tolerant accessions was significantly higher than the intolerant accessions (*p* < 0.001) (Figure 5A and Table S10). A significant difference in K concentration was also observed in stems and roots (*p* < 0.05) (Figure 5B and Table S10), however, for leaves, it was not significant (*p* > 0.05).

## 4. Discussion

### 4.1. Identification of Genomic Regions Conferring Salt Tolerance in Lentil

Salt tolerance is a genetically and physiologically complex trait [26,63] and a severe constraint to lentil yields globally. To improve lentil productivity, the most direct approach is to identify and increase the presence of novel genes and alleles associated with salt tolerance in commercially relevant lentil germplasm. Although, there have been several studies undertaken in lentil describing salt tolerance genetics [18,64], a comprehensive analysis based on diverse germplasm and genome-wide set of SNP markers is limited. As such, the present study was conducted for a better understanding of the salt tolerance in lentil using both advanced genomics and phenomics approaches.

As would be expected between the two GBS pipelines used, tGBS provided a more uniformly distributed genome-wide set of SNP markers for GWAS analysis; as it was designed with known variants selected for even distribution and maximum performance. However, both GBS methods generated over 50,000 genome-wide SNP markers and provided the necessary genotype data to perform GWAS in lentil and were able to identify the genomic regions associated with the trait. Both marker systems pinpointed the major genomic region for salt tolerance on Chromosome 2, while tGBS also identified an additional association on Chromosome 4, which was not detected by GBS-t approach. However, both GBS methods captured this region on Chromosome 4, using the image-based phenotypic screen. The non-overlapping nature of the regions identified on Chromosome 4 is likely due to the lower SNP marker density in the GBS-t method, in the specific region of 395–410 Mbp (approximately half the SNP marker number, Figure S3C). These findings demonstrate that tGBS delivers better-distributed markers that enable a more sensitive approach than the GBS-t method. Therefore, the tGBS assay described here is more likely to be a superior approach for future studies due to its robustness, reliability, and reproducibility. Currently, many researchers based on plants, animals, and human studies aim for targeted-capture-based approaches to sequence specific variant regions or genes of interest selectively to maximize the reliability and robustness of association and GS studies [31,32,35]. However, in the absence of a well-designed tGBS assay, the GBS-t approach performs adequately and would deliver almost comparable results [30,65].

The phenotypic scores used within this study were collected by traditional visual-based screening, using a growth response scale which accounts for multiple factors, including plant appearance, height, greenness, and growth [4,40], and hence is a complex/compound trait for salt tolerance. Therefore, during the marker-trait associations, some of the components can be lost or result in lower associations. This is well exemplified by the region on Chromosome 4, only weakly identified by the tGBS method. However, through undertaking detailed specific trait evaluation, this can be overcome. The green color pixels taken from image-based screening using automated-glasshouse facilities were moderately correlated to the traditional phenotypic score (*r* = 0.55, *p* < 0.0001) [40]. This dataset precisely details a component of the trait and identified significant marker-trait associations on Chromosome 4 using both genotyping methods. The association on Chromosome 2 could be due to the other subcomponents, including plant appearance and growth measured from the traditional screen. This was also in line with other association studies related to salt tolerance, where the high number of quantitative trait loci (QTLs) were identified based on the trait defined [66,67]. Therefore, the combined use of both data sets leads to a more comprehensive dissection of the complex trait and has delivered a greater resource and understanding for genetic improvement for the future. However, the best solution for identifying all these effects on complex traits such as salt stress can be achieved via GS.

### 4.2. Breeding for Salt Tolerance, Haplotypes, and Pedigree Analysis

Over the past two decades, attempts were made to generate salt-tolerant lentil varieties through conventional breeding. However, the understanding of precise chromosome locations/SNP markers for salt-responsive traits, can accelerate germplasm enhancement for salt tolerance either through marker-assisted selection (MAS) or GS-based breeding. From the current study, strong linkage disequilibrium was observed for the major haplotypic region on Chromosome 2 relating to tolerance, for robust marker-trait associations to be available it is now imperative to assess the functional relevance of these clustered SNP markers.

Although the application of MAS is simple, the lack of knowledge and the complete association between marker-trait or salt-responsive genes, and the complex inheritance of the trait can result in the failure of the selective breeding process. Approaches like GS are more powerful, however often multiple traits are necessary to be selected simultaneously to justify the genotyping costs [68]. Although GS requires genome-wide markers to estimate the effects of all loci, the use of prior biological knowledge and prediction tools such as BayesRC have been shown to deliver superior predictions [69]. This approach of functional SNP weighting has been applied in several studies related to animal and plant species, where prior knowledge on loci information was successfully used to predict milk production in dairy cattle [69] and blackleg disease resistance in canola [70]. The data presented in this study relating to salt-responsive traits could now be used as a foundation dataset to build GS-prediction equations for salt tolerance in lentil.

The haplotype identified in salt-tolerant lentil accessions needed to be understood in terms of relatedness and the potential for identity by descent breeding. Pedigree analysis performed on salt-tolerant lentil genotypes identified only two accessions, ILL7685 and ILL1719 as the potential sources of allele contribution to salt tolerance in lentil. Both accessions have the same haplotype (Hap1) and therefore might be closely related. ILL1719 is an Ethiopian lentil landrace that dates from the 1970s [71]. According to a review by Qureshi et al. [72], soil salinity is one of the major abiotic stress factors in Ethiopia; the country being ranked as 7th globally in terms of land area affected with salinity. ILL7685 is a breeding variety from the 1990s [73] and carries a similar haplotype and salt-tolerant trait to ILL1719. These dates could mean that ILL7685 is derived from ILL1719 with some general performance enhancements and was, therefore, more widely used in the lentil breeding program. According to Materne et al. [74], ILL7685 was identified as one of the salt-tolerant and highest-yielding lentil accessions grown under high saline soils (EC = ~ 4 dS/m; pH = ~9.0) at Birchip, Mallee, VIC, Australia. However, due to the complexity of phenotypic-based breeding methods, most industry-leading lentil cultivars have currently lost or have not received genetics related to salt tolerance [59]. Therefore, the current study provides valuable resources to enhance lentil breeding through targeted genomics for salt-tolerant regions that can be applied to improve lentil productivity in Australia and globally.

### 4.3. Potential Candidate Genes and Salt Tolerance Mechanism in Lentil

In the current study, 43 and 1195 candidate genes were identified within the Chromosome 2 and 4 haplotypic regions relating to the trait. A wide array of genes were identified including transporter genes (e.g., *HKT*, auxin influx transporter, ion transporters such as sulfate, potassium and phosphates), kinases (e.g., pyruvate kinase, LRR receptor-like kinase, sucrose non-fermenting 1: SNF1-related kinase, mitogen-activated protein kinases (*MAPKs*), serine/threonine kinase), transcription factors (e.g., *BZIP*, Myb-like transcription factor family protein), as well as other proteins including, transmembrane proteins, vacuolar protein sorting associated protein, *SOS1*, and multiple uncharacterized and hypothetical proteins.

Based on previous studies, most of these transporter genes, kinases, and transcription factors are reported to play an essential role in a salt stress response [1,10,75,76]. However, the importance of them in the salt tolerance mechanism in lentil is not known. The mineral composition analysis identified the tolerant lentil accessions maintained a lower level of Na^+^ in aerial plant tissues but had the same level of K^+^ ion concentration compared to intolerant accessions under salt stress conditions. These observations are different from other salt tolerance studies on unrelated lentil accessions where tolerant genotypes regulate a relatively higher level of K^+^, with proper maintenance of Na^+^ ions [18,19]. Therefore, these findings conclude that the current study has identified a novel source of salt tolerance that could potentially be combined with the alternative mechanism to achieve improved levels of tolerance in lentil.

Mineral analysis in the current study has also shown that the tolerant accessions maintain higher Na^+^ ion concentrations in root tissues than intolerant accessions. Hence, this result implies that either Na^+^ ions absorbed by the plant were actively re-transported to the roots or were held in the root tissues to prevent lethal effects on shoots and leaves. Therefore, once this functional overlay is added to the candidate gene list, the candidate gene that mechanistically agrees with the data is the high-affinity potassium transporter-*HKT*. However, there may be other salt tolerance mechanisms functioning from this region, however, mineral analysis has only statistically confirmed that Na^+^ ions are higher in roots and lower in aerial plant tissues in the plant.

According to Roy et al. [9], *HKT* is involved in regulating Na^+^ ion transportation. In *Arabidopsis thaliana*, the overexpression of *HKT1-1* resulted in more removal of Na^+^ ions from the xylem tissues into the specialized compartments in the root tissues to avoid any lethal effects [75]. The *HKT* gene expression under salt stress has also been reported in several other plant species, including rice [77] and barley [78], where *HKT1;5* was identified as the respective *HKT* family gene responsible for salt tolerance in both of them. Unfortunately, there were no SNP or allele sequences generated for the *HKT* in the current study. Therefore, allele resequencing of tolerant and intolerant lentil accessions through an amplicon-based sequencing approach would be the next step for analysis of the functional nature of the salt tolerance mechanism in lentil. Trait-linked molecular markers have the potential to enhance the efficiency in the selection of superior varieties for salt tolerance using genomic-based breeding. Therefore, the targeted regions identified in this study will further contribute towards GS and modern breeding practices to shorten the cycle time of the Australian lentil breeding program.

## 5. Conclusions

Although high-quality genotypic data was collected from both targeted GBS and exome sequencing approaches, the targeted GBS approach clearly provided superior evenness of coverage and marker numbers that provided good results in this study and will benefit a wide range of future studies in lentil genetics. From the GWAS performed, the significant regions on Chromosome 2 and Chromosome 4 for salt tolerance traits and useful candidate genes identified can now simply be selected for functional studies to generate salt-tolerant germplasm. In addition, the identification of haplotypic blocks and their common ancestry describes well the progress that breeders’ selections have already made. The results generated in this study add to the understanding of salt tolerance mechanisms and will help the breeding programs to make further gains in salt tolerance improvement with significantly reduced timelines.

## Figures and Tables

**Table 1:** Number of SNPs remaining after each filtering step.

Filtering Step	Targeted-GBS (tGBS) Method	Transcriptome-Based GBS (GBS-t) Method
Depth 5	2,043,680	1,614,141
Maximum missing 80 and Q30	457,692	90,493
Heterozygosity 0.2	450,927	85,641
Minor allelic frequency 0.05	57,344	53,186
Final analysis	56,349	52,471

**Table 2:** Favorable alleles observed in significant SNP positions (−log_10_ *p*-value ≥ 5) on Chromosome 2 for phenotypic scores, using two GBS methods.

Position	GBS Method	Alleles	Tolerant		Intolerant	
			Favorable Allele	No. of Accessions	Favorable Allele	No. of Accessions
392,560,939	tGBS	A/G	A	28	G	34
392,619,081	tGBS	C/G	C	26	G	35
392,619,090	tGBS	C/T	T	26	C	35
392,619,094	tGBS	A/T	A	26	T	35
392,619,099	tGBS	A/G	A	26	G	35
392,619,165	tGBS	A/C	C	28	A	35
392,690,695	tGBS	C/T	T	25	C	33
392,690,695	GBS-t	C/T	T	31	C	27
392,690,876	GBS-t	A/G	G	31	A	29
392,692,372	GBS-t	T/G	G	31	T	31
392,692,397	GBS-t	A/G	G	29	A	32
392,695,004	GBS-t	A/G	G	31	A	29
392,695,044	GBS-t	C/T	T	32	C	32
392,695,091	tGBS	A/G	A	23	G	35
392,695,091	GBS-t	A/G	A	32	G	32
392,695,192	tGBS	C/T	T	23	C	35
392,697,101	GBS-t	T/C	C	32	T	32
392,697,852	GBS-t	T/A	A	30	T	32
392,697,951	GBS-t	T/C	C	29	T	32
392,699,727	GBS-t	T/C	C	32	T	31
392,700,661	GBS-t	C/A	A	31	C	31
392,758,269	tGBS	C/T	C	26	T	33
392,767,939	GBS-t	C/A	A	28	C	30
393,426,328	tGBS	G/T	T	26	G	35
393,426,332	tGBS	C/T	T	22	C	35
393,427,373	GBS-t	C/T	T	31	C	30

## Data Availability

The data and detailed results are provided in Supplementary files.

## Supplementary Materials

The following are available online at https://www.mdpi.com/2073-4425/12/3/332/s1, Figure S1: Genetic diversity of the studied lentil population, Figure S2: Model selection for GWAS (A) GLM (without PCA) (B) GLM (only PCA) and (C) MLM (both PCA and Kinship (K)), Figure S3: Comparison of marker-trait association results obtained from traditional phenotypic scores and phenomics screen green color (A) Zoom-in image of traditional phenotypic scores; left: tGBS and right: GBS-t (B) Zoom-in image of phenomics screen green color; left: tGBS and right: GBS-t (C) Comparison of zoom-in images obtained from traditional phenotypic scores (Chromosome 2) and phenomics screen green color (Chromosome 4), using both GBS method, Figure S4: Marker-trait association results for salt tolerance traits. (A) and (B) represent the plots generated for shoot dry weights. (C) and (D) represent the plots generated for projected shoot area. (E) and (F) represent the plots generated for height (G) and (H) represent the plots generated for convex hull (I) and (J) represent the plots generated for compactness (K) and (L) represent the plots generated for non-green color. Plots obtained from tGBS illustrated on the left, while plots obtained from GBS-t method illustrated on the right. The red line indicated the significant threshold level, Figure S5: LD heatmap on Chromosome 2 haplotypic region using SNP markers generated from both GBS methods. Markers derived from each GBS method were highlighted using the colored box. Blue: markers from tGBS method; Green: markers from GBS-t method; and Yellow: markers common to both GBS methods. ‘*’ represents the significant marker positions identified in the current study, Figure S6: Pedigree analysis on tolerant lentil accessions using ILL7685 as the parental source. Salt tolerant accessions were color-coded according to the haplotypes identified in the current study; Red: Hap1, Green: Hap2, and Yellow: Hap1/2. The lentil accession that failed to have sufficient pedigree information was highlighted in the black square, Figure S7: Pedigree analysis on tolerant lentil accessions using ILL1719 as the parental source. Salt tolerant accessions were color-coded according to the haplotypes identified in the current study; Red: Hap1, Green: Hap2, and Yellow: Hap1/2. The lentil accession that failed to have sufficient pedigree information was highlighted in the black square, Figure S8: Variation in micro- and macro elements in different tissue types collected from tolerant and intolerant lentil accessions (A) Ca (B) Mg (C) P (D) Cu (E) S (F) B (G) Al (H) Fe (I) Zn (J) Mn, Table S1: Phenotypic data used in this study, Table S2: Flanking sequencing information of the tGBS design, Table S3: List of high-confidence SNP markers identified from tGBS method, Table S4: List of high-confidence SNP markers identified from GBS-t method, Table S5: Number of variants identified for each chromosome, using two GBS methods, Table S6: The summary statistics of significant marker-trait positions, Table S7: Candidate genes identified from significant haplotypic regions, Table S8: Haplotypes variation in tolerant and intolerant lentil accessions, Table S9: Input data for pedigree analysis, Table S10: Pairwise comparison of Na and K for salt tolerance in lentil.
