# Supplementary figures and images for: Application of Genomics to Understand Salt Tolerance in Lentil
Source: Genes (Basel). 2021 Feb 25;12(3):332. doi: 10.3390/genes12030332 (PMC7996261; doi:10.3390/genes12030332)

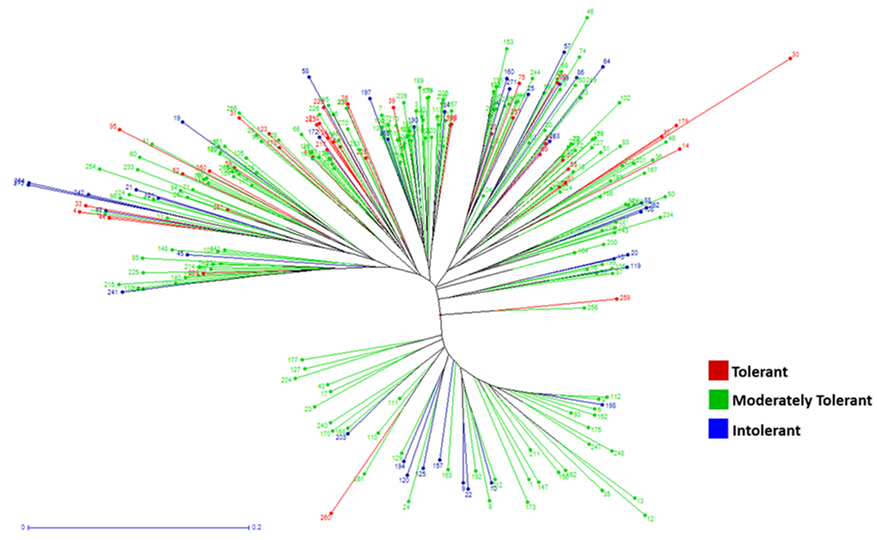

Supplement: Supplementary file 1 [file genes-12-00332-s001.zip › Supplementary files/Figure S1.tif]

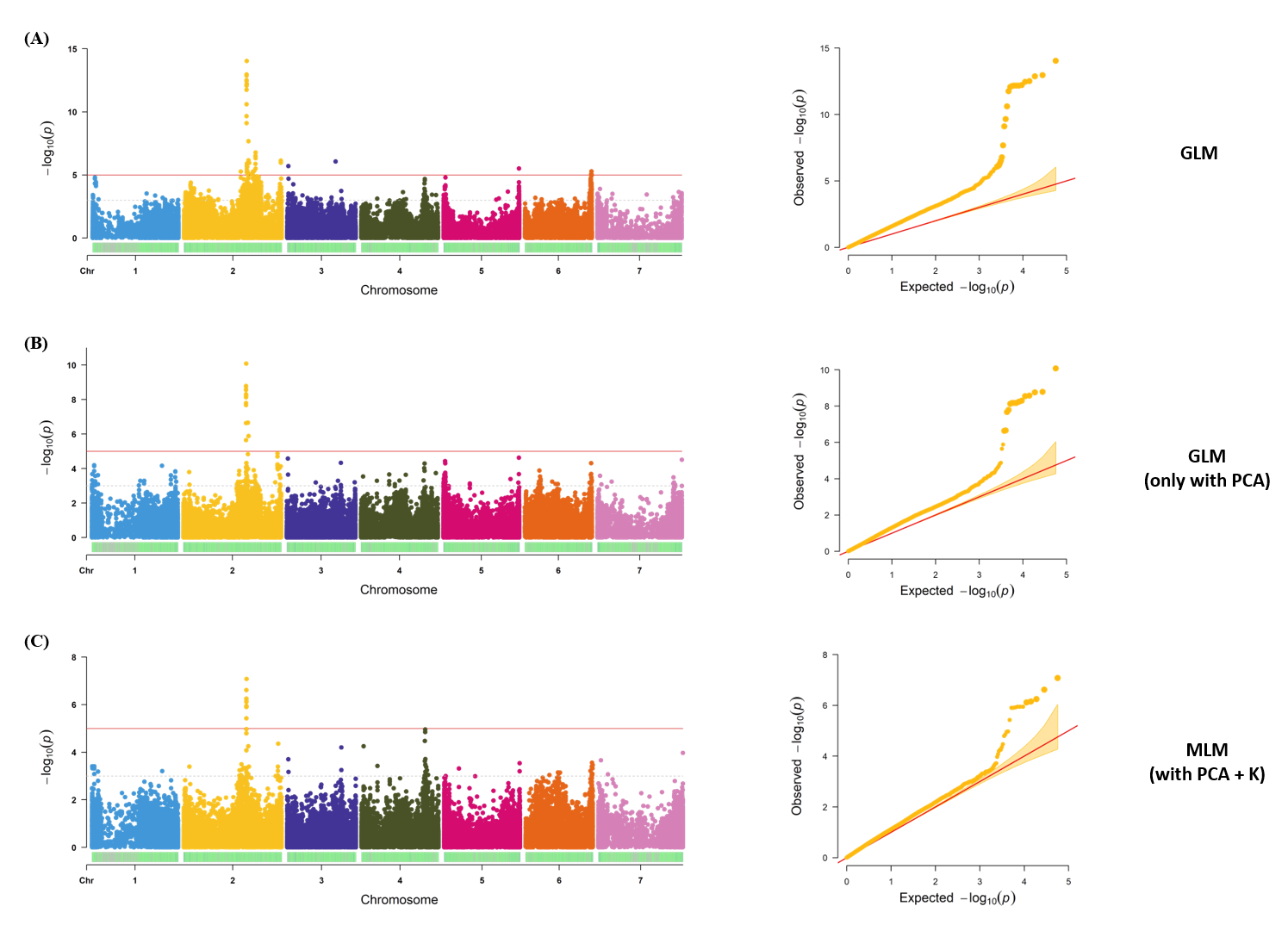

Supplement: Supplementary file 1 [file genes-12-00332-s001.zip › Supplementary files/Figure S2.tif]

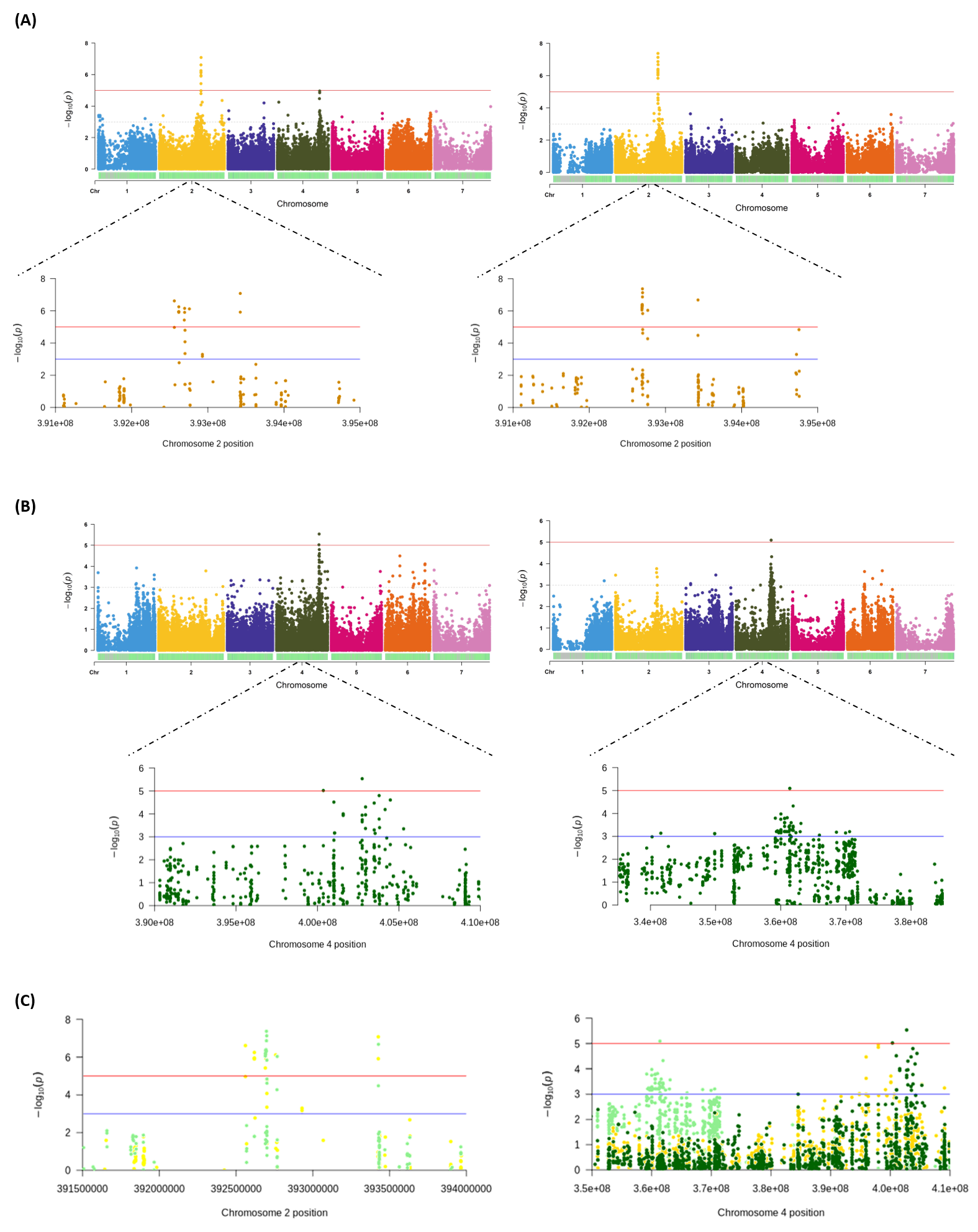

Supplement: Supplementary file 1 [file genes-12-00332-s001.zip › Supplementary files/Figure S3.tif]

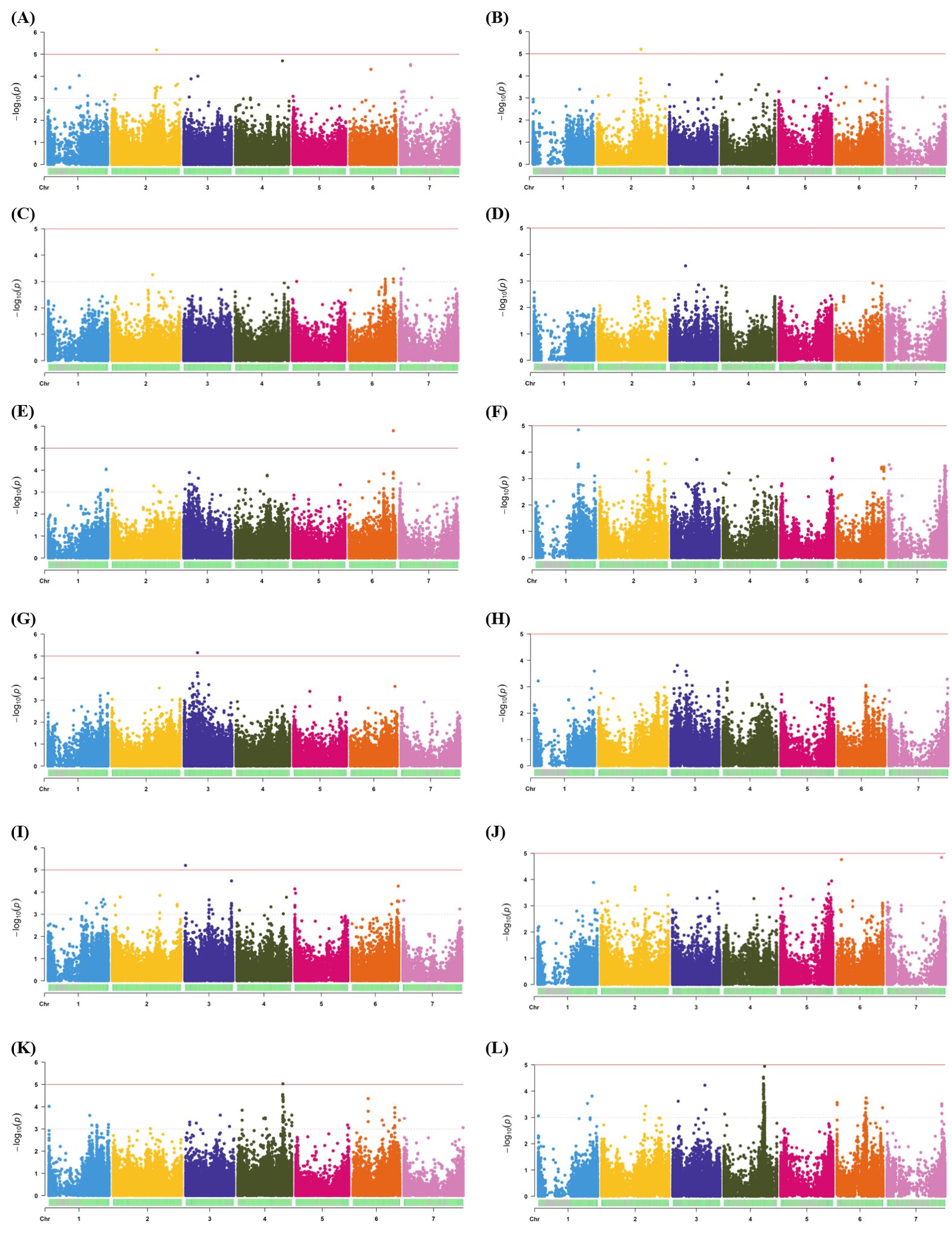

Supplement: Supplementary file 1 [file genes-12-00332-s001.zip › Supplementary files/Figure S4.tif]

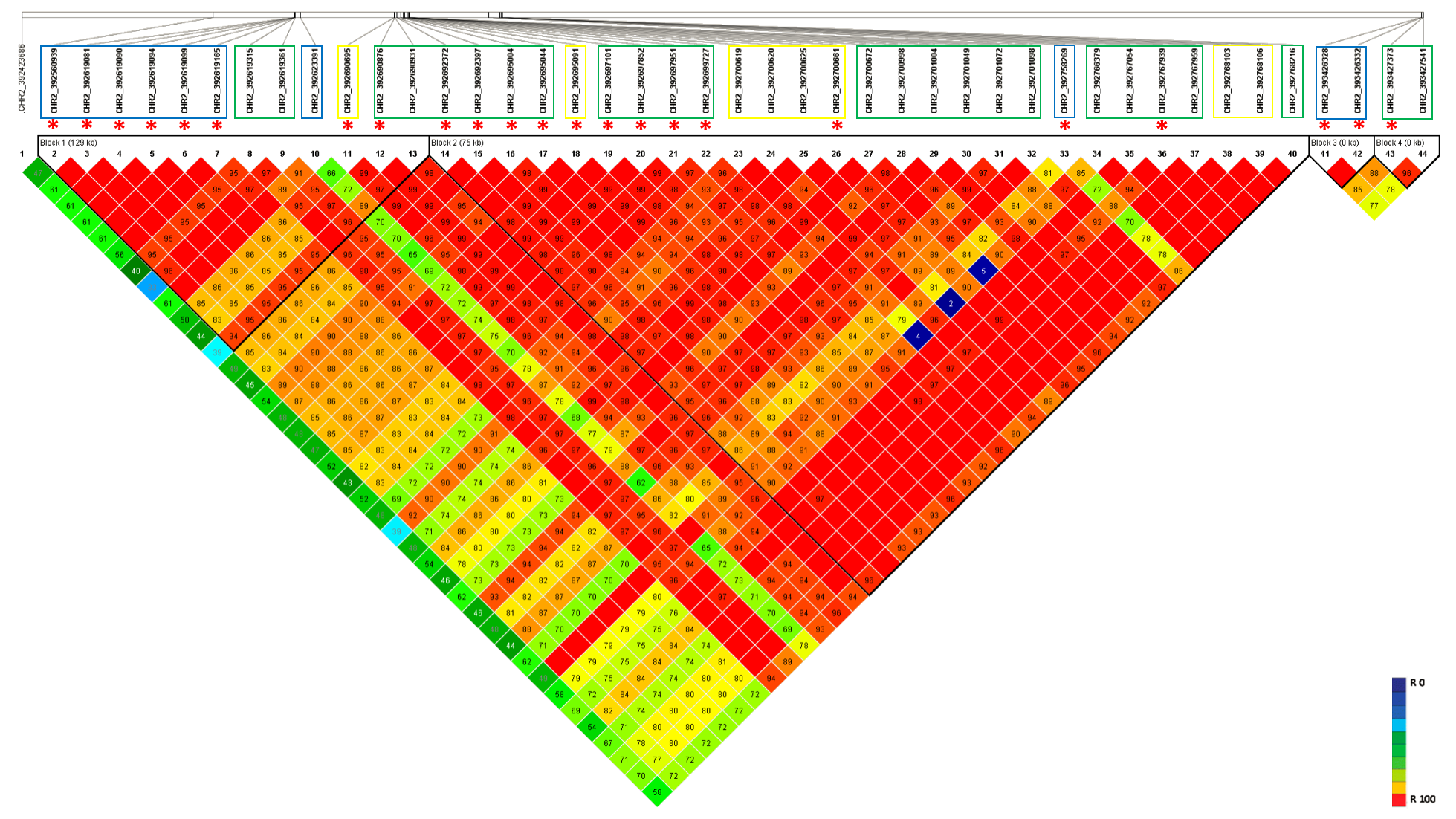

Supplement: Supplementary file 1 [file genes-12-00332-s001.zip › Supplementary files/Figure S5.tif]

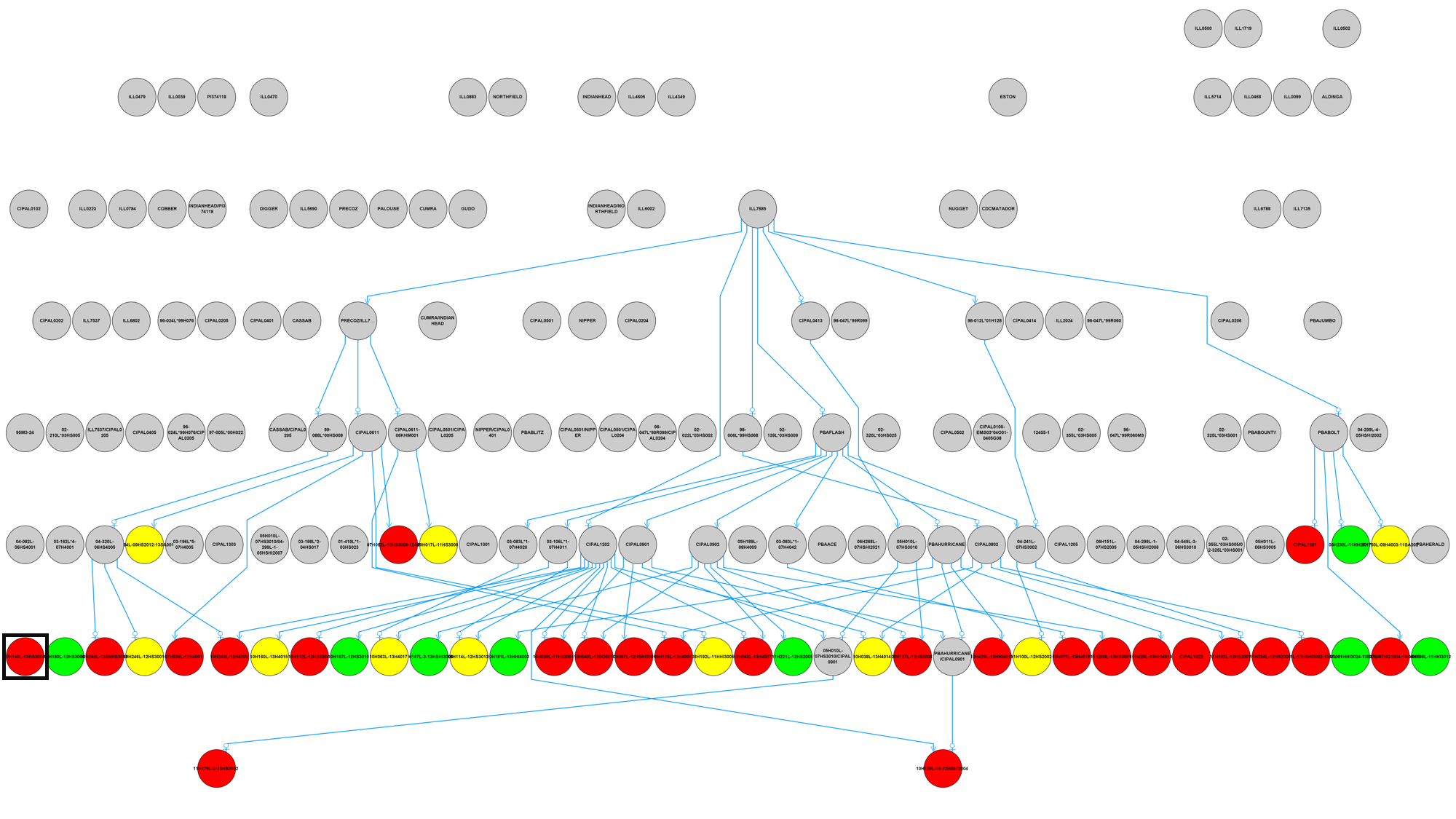

Supplement: Supplementary file 1 [file genes-12-00332-s001.zip › Supplementary files/Figure S6.png]

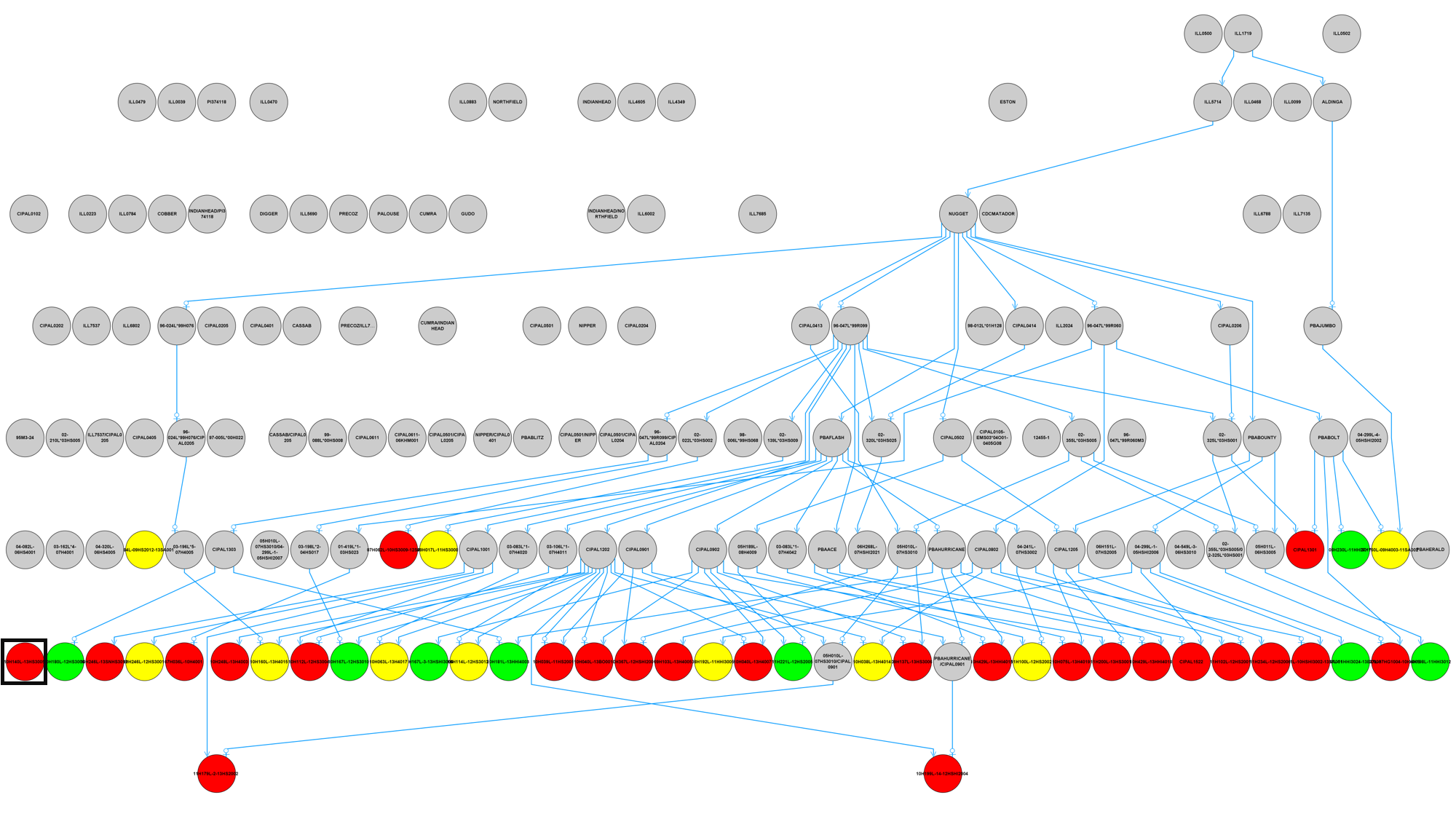

Supplement: Supplementary file 1 [file genes-12-00332-s001.zip › Supplementary files/Figure S7.png]

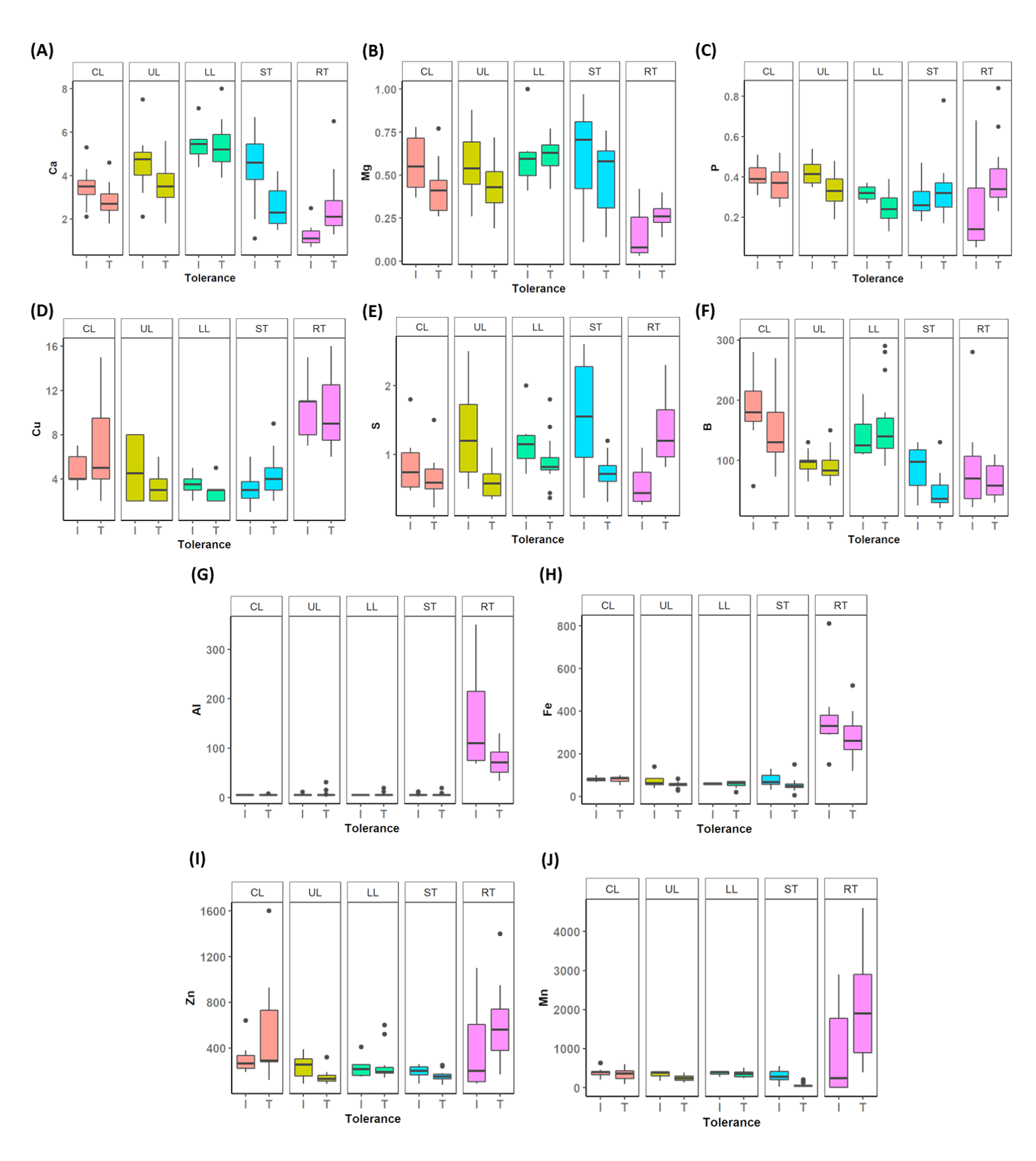

Supplement: Supplementary file 1 [file genes-12-00332-s001.zip › Supplementary files/Figure S8.tif]
